# On the Application of Hot Flour in Erysipelatous Inflammations, and Cutaneous Eruptions:—Extracted from a Memoir of Alphonse Leroy, Professor in the School of Medicine at Paris

**Published:** 1799-04

**Authors:** 


### *On the Application of hot Flour in Erysipelatous Inflammations, and cutaneous Eruptions:—extracted from a Memoir of* Alphonse Leroy, *Professor in the School of Medicine at Paris.*

IN the first number of this Journal, page 84, we mentioned the *external application of hot flour*, as a new remedy, which has been successfully employed in Paris, by the strong recommendation of Leroy. The importance of such a discovery, and the celebrity of its author, demand a more particular account of its application, as well as its effects.

The first case related by Citizen Leroy, is that of a woman to whom he was called, in 1784, afflicted with an erysipelas in her face, accompanied with ulcers. Having formerly observed, that lotions, cataplasms, decoctions of elder, and fat substances, were often hurtful in erysipelas, he thought proper to forbid the use of them to his patient, and prescribed the application of wheat-flour, heated in a skillet. A surgeon, however, who came in immediately after, strongly condemned this remedy, and persuaded the patient to apply a poultice of bread and milk. Next day, Citizen Leroy found her face in a deplorable state; the eyes swelled, and running with water, and the power of vision apparently lost. This dreadful effect from the application of a cataplasm, for only twelve hours, still more confirmed him in the opinion, that *desiccatives*, such as hot meal, and hot ashes, were most proper in many cutaneous diseases.

In

In 1791, he was called to the female citizen DANGIVILLER, when he found in a miserable situation. The disorder had begun with large ulcers on her arms, legs, and feet, filled with a clear aqueous humour, which turned into white matter, resembling that in the small-pox, and threatened to spread over the whole body. Her attendants had placed her on a couch, and covered the ulcerations with cataplasms. The fever was broke out, and there began to appear little black mortified spots. Not thinking it proper to make too rapid a transition from moistening to drying applications, Citizen Leroy prescribed, for twenty-four hours, a paste between two pieces of fine linen, to be changed every six hours: next day, he ordered the parts affected to be covered with dry and hot flour. This remedy was immediately related and ridiculed at court. The physicians amused their friends afflicted with similar complaints, by passing witticisms upon the flour-bath; but they did not long enjoy their augh, for Leroy's patient quickly recovered.

In 1795, Leroy was called to a woman, who, during her imprisonment, had made use of great quantities of vinegar, as a preventive against the jail fever. When he saw her, she had been five weeks confined to her bed, covered from head to foot with suppurating ulcers. Under a large pellicle he observed a watery humour, which thickened into pus. Notwithstanding the assiduous attention of Citizen OBERLIK, an eminent physician at Noyon, who had ordered dry rags and lint to be applied to the sores, and seldom poultices, the disease increased, and the patient was reduced nearly to the last extremity. Citizen Leroy directed the suppurating parts to be covered with hot flour; he also ordered her a decoction of bark, to be taken several times a day. He prescribed weak broth of veal, or fowl, which they had not ventured to give her, for fear of increasing putrefaction, a very generally adopted maxim in medicine, but too often fatal in its effects. In a short time, however, his patient recovered.

Last summer he was called to Citizen DELASSALE, aged 65 or 66, who had, for several years, been afflicted with a succession of disorders. He was threatened with dropsy in his breast; then attacked with a catarrh, which was succeeded by an excessive hæmorrhage of the lungs. At length, having recovered from these different diseases, he was troubled with ulcers on his back, shoulders, and breast. He would have appeared like a person flayed, if the pellicles which covered the ulcers had been taken off. The Professor caused him to be covered with linen cloths, filled with hot flour, powdering at the same time the suppurating parts, which began to emit a fetid smell. This, with the extract of bark, weak broth, and light food, completed his cure. Notwithstanding the fever, he prescribed the broth, considerconsidering moist and sweet articles of food necessary in debilitating fevers, and which are very different from those of an inflammatory kind.

Very lately, he was called to the chambermaid of the female Citizen Merlin, wife of the present Director. Her disorder was an erysipelas in the face and neck. They had been bathed with elder-water, and assumed a serious appearance, the fever commencing with drowsiness. Cit. Le Roy prohibited all wet applications, and substituted dry flour, keeping her body open, which soon brought her out of danger.

The same remedy was successively employed by Cit. Le Roy in the ring-worm, or tetters in the face, which seemed almost incurable. This regimen is also applicable in swellings of joints, to which the watery or moist applications are obviously injurious.

Thus (concludes Le Roy) dry and hot flour, hot-ashes, earths, aromatic and other vegetable powders, combined with heat, are powerful remedies. The watery element appears only proper to cure the internal disorders of the animal economy. These observations he hopes will excite the attention of practitioners to the opposite element, viz. dry and earthy substances.

Whatever merit may be due to Professor Le Roy for his useful suggestions, he is not entitled to that of originality; nor will any experienced practitioner agree with him, that *dry* applications are useful in *all* stages of erysipelas, and other cutaneous, as he seems inclined to maintain.

Dr. Unzer, of Hamburgh, as well as Dr. Gesenius, of Nordhausen, mention in their several works, a powder which has long been used in Germany, as a powerfnl discutient in erysipelas, arising from external injury, if the complaint be not merely a symptom or crisis of another more important disease. This powder consists of a mixture of dry *flour* with aromatic vegetables, a small addition of sugar of lead, instead of which, if occasion requires, camphor is sometimes substituted. If the ingenious Le Roy had pointed out, with accuracy aud precision, the particular habits, temperaments, constitutions, and other circumstances which deserved to be noticed in his patients; if he had informed us of the predisposing and proximate causes which gave rise to the virulent cutaneous disorders he has cured; and lastly, if he had described nosologically the particular species of cutaneous eruptions, together with the character and type of the fever which accompanied the complaint in each individual patient—the medical world would have been infinitely more indebted to him for such information, than for the most favourable accounts he could give of *general*, or, to speak more freely, *empirical practice*.

