# Errata

**Published:** 1799-05

**Authors:** 


—— 154 (*and* 84 *of No. I.*), *in the account of the cure of eryfipelatous inflammations, and other cutaneous difeafes, by Profeffor Leroy, the word* flour *is uniformly fpelt* flower; *becaufe the former mode of writing it, which prevails at prefent, could not be found either in Johnfon's or Bailey's Englifh Dictionaries.*

